# EMPOWER: A Feasibility Study of a Remote and In-Person Integrative Intervention for Caregiver Support

**DOI:** 10.1093/geroni/igaf122.2650

**Published:** 2025-12-31

**Authors:** Katie Trainum, Rosa Schnyer, Bo Xie

**Affiliations:** University of Pennsylvania, Philadelphia, Pennsylvania, United States; The University of Texas at Austin, Austin, Texas, United States; The University of Texas at Austin, Austin, Texas, United States

## Abstract

Family caregivers face substantial caregiving-related burdens, needing supportive interventions, particularly those incorporating self-care strategies that can be delivered remotely with broad accessibility. We developed EMPOWER (Engage your Mind and Body to Promote your Own Wellness, Energy and Relaxation), an intervention incorporating complementary and integrative strategies like breathing exercises, Tai Chi, and self-acupressure. In Fall 2024, we conducted four sessions (3 via Zoom; 1 in-person) to evaluate EMPOWER’s feasibility. Following recruitment challenges, including scammers in the first two Zoom sessions, the final sample included 8 caregivers (7 female; age range 55-90; mean = 68.38; 6 remote, 2 in-person). Each 90-120-minute session involved instruction and practice led by an experienced clinician. Participants received program handouts and audio recordings for at-home practice. Two weeks later, 75% (n = 6) completed a post-intervention questionnaire online or over the phone, including 2 from the in-person session and 4 from the third Zoom session. Data were analyzed using content analysis and paired t-tests. T-tests revealed no significant changes in caregiver burden, stress, anxiety, or quality of life from pre- to post-intervention across all groups. Content analysis of qualitative data suggested improved relaxation, stress management, empowerment, and regular practice. Participants also reported challenges such as technological barriers, physical limitations, and time constraints in both session formats. These findings support the feasibility of delivering EMPOWER remotely and in-person for caregivers. Future work should refine the intervention to overcome the identified challenges, incorporate a mobile application for shorter, more accessible sessions, and test with a larger sample.

